# Patients’ preferences for primary health care – a systematic literature review of discrete choice experiments

**DOI:** 10.1186/s12913-017-2433-7

**Published:** 2017-07-11

**Authors:** Kim-Sarah Kleij, Ulla Tangermann, Volker E. Amelung, Christian Krauth

**Affiliations:** Institute for Epidemiology, Social Medicine and Health Systems Research, Hannover Medical School, Carl-Neuberg-Str. 1, 30625 Hanover, Germany

**Keywords:** Preferences, Primary health care, Discrete choice experiment, Systematic review

## Abstract

**Background:**

Primary care is a key element of health care systems and addresses the main health problems of the population. Due to the demographic change, primary care even gains in importance. The knowledge of the patients’ preferences can help policy makers as well as physicians to set priorities in their effort to make health care delivery more responsive to patients’ needs. Our objective was to describe which aspects of primary care were included in preference studies and which of them were the most preferred aspects.

**Methods:**

In order to elicit the preferences for primary care, a systematic literature search was conducted. Two researchers searched three electronic databases (PubMed, Scopus, and PsycINFO) and conducted a narrative synthesis. Inclusion criteria were: focus on primary health care delivery, discrete choice experiment as elicitation method, and studies published between 2006 and 2015 in English language.

**Results:**

We identified 18 studies that elicited either the patients’ or the population’s preferences for primary care based on a discrete choice experiment. Altogether the studies used 16 structure attributes, ten process attributes and four outcome attributes. The most commonly applied structure attribute was “Waiting time till appointment”, the most frequently used process attribute was “Shared decision making / professional’s attention paid to your views”. “Receiving the ‘best’ treatment” was the most commonly applied outcome attribute. Process attributes were most often the ones of highest importance for patients or the population. The attributes and attribute levels used in the discrete choice experiments were identified by literature research, qualitative research, expert interviews, or the analysis of policy documents.

**Conclusions:**

The results of the DCE studies show different preferences for primary health care. The diversity of the results may have several reasons, such as the method of analysis, the selection procedure of the attributes and their levels or the specific research question of the study. As the results of discrete choice experiments depend on many different factors, it is important for a better comprehensibility of the studies to transparently report the steps undertaken in a study as well as the interim results regarding the identification of attributes and levels.

**Electronic supplementary material:**

The online version of this article (doi:10.1186/s12913-017-2433-7) contains supplementary material, which is available to authorized users.

## Background

According to the World Health Organization’s (WHO’s) declaration of Alma-Ata, primary care is a key element of health care systems. As it addresses the main health problems in the community and often serves as the patients’ first level of contact with the health care system, primary care is highly important to all citizens [1]. Primary health care encompasses different curative and preventive services, such as diagnosis and treatment of chronic and acute conditions, and patient education concerning the major health problems. Furthermore, primary care traditionally is health care which, in the first place, focuses on the needs of the patients [2]. As most of the Western populations are continuously aging and, hence, the burden of chronic conditions is increasing, primary care even gains in importance. The knowledge of the patients’ preferences can help policy makers as well as physicians to set priorities in their effort to make health care delivery more responsive to patients’ needs [3]. Moreover, the patients’ satisfaction positively influences their compliance with the treatment [4]. As many countries face a shortage of general practitioners (GPs) in rural and remote areas, the maintenance of an adequate primary care provision is a central task of health care systems and therefore a highly important subject for health policies. In order to guarantee an adequate, needs-based medical supply strategies, such as new and innovative models of care are needed. Philips et al. [5] for example state that in many European countries an increasing number of patients is using emergency rooms for less urgent problems in out-of-hours situations. If policy makers attempt to address these problems and to reorganize out-of-hours care more efficiently, they need to know patients’ preferences. If the reorganization does not take into account the patients’ needs and preferences, patients probably would not use them and continue visiting emergency rooms. Thus, it is of high relevance for the future organization of primary care and the introduction of new models of care to know the population’s preferences for different aspects of primary care.

Preferences for medical care can be defined as “statements that indicate the importance of specific aspects of clinical behavior of care providers or the organization of care” ([3], p. 1573). They indicate what should happen and they differ from the concepts of expectations, experiences and satisfaction. The latter can be described as the assessment of the care received, i.e. the assessment of the experiences [2]. Expectations are determinants of satisfaction as well. While predictive expectations describe what people actually believe will happen in the future, ideal expectations are desires connected to an idealistic state of beliefs [6]. Thus, ideal expectations are more abstract than preferences.

The discrete choice experiment (DCE) is a common technique to elicit preferences for health care services or technologies. DCEs are based on Lancaster’s [7] theory according to which the utility of goods or services is determined by different characteristics, called attributes, that characterize the good or service. Each attribute has different specifications, so called attribute levels. In a DCE, a good or service is described based on changing combinations of attribute levels and participants are asked to choose out of two or three different options the one, they prefer. The choices over a number of alternatives can then be analyzed to calculate the relative importance of the attributes. It is assumed that respondents take into account all information provided and then select the alternative which provides the highest utility to them [7]. Changes in the attribute levels can alter the preferred choice alternative of participants [8]. In addition to Lancaster’s theory, discrete choice experiments are based on the random utility theory (RUT) [9, 10]. In contrast to the classic consumer theory, the RUT states that individual choice behavior is probabilistic rather than deterministic. Thus, the utility of a good or service can be divided into an explicable, systematic component and a non-explicable, random component. The latter can for example be due to unobserved preference variation or measurement error, [11].

There are several discrete choice experiments focusing on patient or public preferences for primary care and GPs. Those DCEs include attributes like “Waiting time till appointment” or “Length of the consultation”. The levels for the attribute waiting time could be “same day”, “one day” and “two days”, for example. If “Price” is one of the attributes of the DCE, it is possible to calculate the willingness to pay for the other attributes. As a DCE is an attribute-based method of measuring preferences, the identification of appropriate attributes is an essential task [2]. Attributes should be important to the respondents on the one hand and relevant to policy makers on the other hand [12]. Therefore, the identification of attributes should always be supported by evidence derived from the literature and/or from qualitative research [13].

The aim of this systematic review is (1) to provide an overview of the attributes and attribute levels used in the discrete choice experiments and of how they were selected and (2) to reveal which attributes of primary care are most important for patients and the population.

## Methods

### Eligibility criteria and search strategy

We conducted a systematic review of published studies reporting stated preferences, particularly discrete choice experiments for primary health care in OECD countries (see Additional file 1 for the review protocol). For obtaining a recent overview of the literature, we searched for studies published in the last 10 years (2006–2015). Electronic searches of relevant databases were conducted on December 7th 2015. The search included (1) terms related to primary care and (2) terms related to preferences and discrete choice experiments, respectively. Search terms were: (“patient* preference*” OR “patient* priorities” OR “public preference*” OR “discrete choice” OR “DCE” OR “conjoint analysis” OR “stated preference*”) AND (“primary care” OR “general practitioner*” OR “GP*” OR “family doctor*” OR “family physician*” OR “family medicine*”) (see Additional file 2 for a detailed presentation of the search strategy). The search was conducted in the databases: PubMed, Scopus and PsycINFO; further literature was added from an additional hand search in the reference lists of the included articles. Titles, abstracts and full-texts of the identified studies from the search strategy were screened for relevance by two of the authors independently (KSK, UT) and ambiguous cases were discussed.

### Inclusion and exclusion criteria

Discrete choice experiments were included if they were published in English and if either the population or patients were asked about their preferences for different aspects of primary health care in general were included. There were no constraints about age, sex or origin of the participants. All types of survey methods were included: face-to-face, telephone, postal, and online surveys. Furthermore, there were no limitations according to the aspects or attributes of primary care, as long as they were relevant for medical care.

Studies were excluded if they only focused on specific conditions because the objective of the literature review was to cover a broad range of attributes that are relevant to all patients, not only patients with specific diseases. Furthermore, we excluded studies that exclusively focused on end-of-life or palliative care or on shared-decision making because they do not refer to primary care in general but focus on an issue that we think is only one aspect or attribute of primary health care.

### Outcomes and comparison of results

The purpose of this review was to identify the attributes and attribute levels used in DCE studies and to provide an overview of the aspects of primary care which are most important to patients and the population. To that end, we divided the identified attributes into the three dimensions “structure”, “process” and “outcome”. These dimensions are based on Donabedian’s model for quality of health care [14, 15] and are appropriate to group the wide range of primary care attributes and to have a closer look on what dimensions of health care are most important for the respondents when choosing primary care conditions. The dimension “structure” refers to objective parameters such as material resources, personal resources and organizational structure. “Process” includes all activities taking place while giving and receiving care, such as diagnosis, prescription and interpersonal aspects. The dimension “outcomes” denotes the effects of health care delivery, including improvements in the patients’ knowledge and changes in their behavior, on the health status of patients [15].

Beyond that, we aimed at giving an overview of how the attributes and attribute levels were selected by the researchers and why they were determined to be relevant. In general, attributes used in a DCE should cover a range that may be relevant to subjects, even if the levels are hypothetical [13]. According to Lancsar and Louviere [11] or Bridges et al. [13] it is good research practice if inclusion or exclusion of potential attributes and levels is based on literature reviews and qualitative research, such as focus group discussions or semi-structured interviews with samples of relevant persons and/or experts. Therefore, we examined if the selections of attributes were based on one or several of these techniques. To extract the data and compare the results, we tabulated the findings for each study.

## Results

### Study selection

**Fig. 1:**
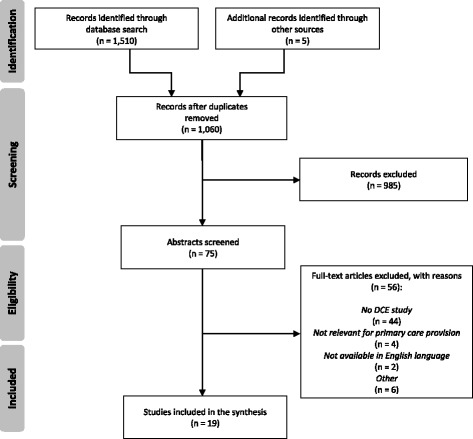
Flow diagram

The search strategy resulted in 1515 findings (Fig. 1): 827 through Scopus, 482 through PubMed, 201 through PsycINFO, and five through the additional hand search. Four hundred fifty-five duplicates were removed and after the subsequent screening of titles, abstracts and full-texts, 19 publications met the inclusion criteria and were thus included in our analysis.^1^ Most studies were conducted in England [16–24]. Other studies were conducted in Scotland [25, 26], Italy [27, 28], Denmark [29], Sweden [30], and the United States [31, 32]. One multinational study [33] was conducted in Germany, United Kingdom and Slovenia. A further study [4] did not indicate the country; it was conducted in a ‘Western European city’. Most of the publications focus on primary care consultations in general. Others consider out-of-hours services, appointment bookings or the role of prescribing pharmacists. One study examines nurse-led versus doctor-led primary care and another publication focuses on retail clinics.

### Study and sample characteristics

Table 1 summarizes the study and sample characteristics. Participants were patients in ten of the articles and the population in eight of the studies. About three quarters of studies used self-complete discrete choice experiments and the number of attributes varied from three to seven, with a mode of four attributes (*n* = 6 studies). The respondents were faced with a maximum of eight different choice sets in most surveys. The sample size was <500 in six and >1000 in seven of the studies. Eight studies had a response rate between 40% and 60%. Three studies reported a response rate of less than 40% and two studies of more than 60%. In these two studies [17, 33] the questionnaires were handed out in a general practice. While one was a self-complete questionnaire, the other was an interviewer-administered survey. Furthermore, some of the studies tested for the influence of sociodemographic and disease-specific characteristics on the preferences [17, 19, 23, 25, 28, 31].

**Table 1:** Study and sample characteristics

	Number of studies (%)
Sample	
Patients	10 (55.6%)
Population	8 (44.4%)
Administration of survey	
Self-complete questionnaire	13 (72.2%)
Interviewer administered	3 (16.7%)
Computerized interview	2 (11.1%)
Number of attributes	
3 attributes	3 (16.7%)
4 attributes	6 (33.3%)
5 attributes	3 (16.7%)
6 attributes	5 (27.8%)
7 attributes	1 (5.6%)
Number of choice tasks per respondent	
8 or less choices	12 (66.7%)
9 – 16 choices	4 (22.2%)
Not reported	2 (11.1%)
Sample size	
< 500	6 (33.3%)
500 – 1000	5 (27.8%)
> 1000	7 (38.9%)
Response rate	
< 40%	3 (16.7%)
40% – 60%	8 (44.4%)
> 60%	2 (11.1%)
Not calculated/reported	5 (27.8%)

### Attributes and levels of primary care

A crucial aspect of discrete choice experiments is the selection of attributes and associated levels that adequately outline the health service a study focuses on. As there are no accurate rules to determine attributes and levels presented in a DCE, we examined how they were selected by the researchers.

Table 2 lists the different methods used by the researchers to determine the appropriate attributes and attribute levels. The most frequently used methods, which are i.a. recommended in the Conjoint Analysis-Checklist published by the International Society for Pharmacoeconomics and Outcomes Research (ISPOR), are a literature research or a systematic literature review (*n* = 10 studies) and qualitative research, such as focus groups or semi-structured interviews (*n* = 8 studies). Other options to identify attributes are discussions with experts (*n* = 3), an analysis of policy documents (*n* = 2) or selection of attributes based on attributes used in former studies (*n* = 3). There are some studies that used several of these methods to identify relevant attributes. Gerard [17] for example conducted seven semi-structured interviews with general practice patients and searched for policy documents and literature to define attributes and levels. In addition to a literature search [2] Cheraghi-Sohi et al. [16] performed qualitative research in the form of the ‘think aloud’ technique to verify the comprehension of the potential attributes. Overall, 11 studies (61.1%) used two or more of these methods to identify attributes and attribute levels (mostly literature review and qualitative research), five studies (27.8%) used one of these methods and two did not report on the identification of attributes.

**Table 2:** Methods to identify attributes and attribute levels

Identification method	No. of studies (%)	
Literature research/review	12	(66.7%)
Qualitative research	10	(55.6%)
Other studies	2	(11.1%)
Policy documents	2	(11.1%)
Experts (GPs)	3	(16.7%)
Nothing reported	2	(11.1%)

Sum is greater than 18 and percentage does not sum up to 100%, because some studies used multiple methods to identify attributes and levels

Table 3 provides an overview of all attributes included in the identified studies and of how many studies used them. As previously described, the attributes are clustered into the dimensions “structure”, “process” and “outcome”. Overall the studies used 18 different structure attributes, 10 process attributes and 3 outcome attributes. Summed up, 52 structure attributes, 27 process attributes and 4 outcome attributes were used. That makes 2.9 structure attributes, 1.5 process attributes and 0.3 outcome attributes per study, on average. The most commonly used structure attribute was “Waiting time till appointment”. Other attributes of this dimension which were frequently used are “Care provider/care setting”^2^ (*n* = 8), and “Waiting time in the practice” (*n* = 6). Among the process attributes, the one most commonly used was “Shared decision making / professional’s attention paid to your views” (*n* = 6). Only a limited number of studies applied outcome attributes. “Chance of receiving the ‘best’ treatment” was used twice and “Chance contact relieves anxiety” as well as “Likelihood of having illness cured” were each used in one study. The levels of the mentioned primary care attributes vary widely. Some attributes like “Care provider” have quite similar levels across the DCE studies (doctor versus nurse practitioner or practice nurse or primary care team), other attributes like “Waiting time till appointment” have very diverse levels (same day, 1 day, 2 days, 3 days, 4 days….10 days). All levels and associates attributes are shown in Table 4.

**Table 3:** Dimensions and attributes

	No. of studies using attribute
Structure attributes (*n* = 18)	
Waiting time till appointment	12
Care provider / Care setting	8
Waiting time in the practice	6
Opening hours	4
Price	4
Convenience of the appointment	3
Individual choice of GP / care provider	2
Technical equipment / Diagnostic facilities	2
Waiting time on the telephone	2
Distance to practice	1
How well practice knows services in neighborhood	1
Informed of expected waiting time	1
Knowledge of how to access the service	1
Method of payment	1
Practice meets specific health needs	1
Price for the drug	1
Time spent travelling and waiting	1
Type of contact	1
Process attributes (*n* = 10)	
Shared decision making / professional’s attention paid to your views	6
Continuity of health professional / physician’s knowledge of the patient	5
Information and explanation (on medicines / treatment / problem)	5
Length of consultation	5
Biopsychosocial perspective	1
Doctor listens	1
Help offered by professional	1
Physician’s interpersonal manner	1
Scope of health review	1
Thoroughness of physical examination	1
Outcome attributes (*n* = 3)	
Receiving the ‘best’ treatment	2
Chance contact relieves anxiety	1
Likelihood of having illness cured	1

One study [31, 32] used the attribute “acuteness”. This attribute does not match one of the dimensions and is therefore excluded

**Table 4:** Overview of attributes, levels and results

No.	Authors	Year	Country	Study objective / aim of the study	Attributes (Level)	Most important attribute	Dimension of most important attribute
1	Ahmed, Fincham	2010 & 2011	USA (Georgia)	Retail clinics: investigate the effects of cost of care and waiting time on care-seeking decisions at retail clinics or physician offices	1. Price ($59; $75), 2. Appointment wait time (same day; 1 day or longer), 3. Care setting–clinician combination (nurse practitioner in retail clinic; physician in private office), and 4. acute illness (urinary tract infection [UTI]; influenza)	appointment wait time	structure
2	Caldow et al.	2006	Scotland	Models/provider of primary care: investigate patient opinion about the provision of nurse-led vs. doctor-led primary health care in the treatment of minor illness	1. Who you see (doctor vs. practice nurse), 2. Waiting time till appointment (no waiting time/2 days/4 days/8 days), 3. Length of consultation (5 min/10 min/20 min/30 min), 4. Continuity of health professional (yes vs. no), 5. Likelihood of having illness cured (75%/80%/85%)	who you see	structure
3	Cheraghi-Sohi et al.	2008	England (Manchester)	Primary care (consultations) in general: assess patients’ priorities for a range of attributes of primary care consultations	1. Number of days wait for an appointment (same day/next day/2 days/5 days), 2. Cost of appointment to patient (£0,/£8/£18/£28), 3. Physician’s knowledge of the patient (the doctor has access to your medical notes and knows you well vs. the doctor has access to your medical notes but does not know you), 4. Patient perspective (the doctor is interested in your own ideas about what is wrong vs. the doctor is not interested in your own ideas), 5. Biopsychosocial perspective (the doctor asks about your social and emotional well-being as well as physical symptoms vs. the doctor asks about your physical symptoms only), 6. Shared decision making (the doctor involves you in decisions about treatment vs. the doctor does not involve you)	physician’s knowledge of the patient	process
4	Gerard	2008	England	Appointment booking: determine the relative importance of factors that influence patient choice in the booking of general practice appointments for two health problems	1. Day of appointment (same day/next day/5 days later/10 days later), 2. Professional person (nurse/doctor, any available/doctor of choice), 3. Time of day of appointment (inconvenient vs. convenient), 4. Length of appointment (10 mins/20 mins)	professional person	structure
5	Gerard et al.	2006	England	Out-of-hours services: Establish which generic attributes of general practice out-of-hours health services were important to members of the public	1. Time to making initial contact (1 min/ 5 min/10 min/15 min), 2. Time waiting for advice or treatment (5 min/20 min/1 h/5 h), 3. Informed of expected waiting time (no vs. yes), 4. Type of contact (by telephone/in person), 5. Professional person (specially trained nurse vs. doctor), 6. Chance OOH contact relieves anxiety (50% vs. 90%)	professional person	structure
6	Gerard et al.	2012	England	Prescribing pharmacists: quantify patients’ preferences for new pharmacist independent prescribing services in general practice	1. Length of consultation (5 min/10 min/20 min), 2. Professional’s words and explanations about your medicines (difficult to understand vs. easy to understand), 3. Attention paid by professional to your views about medicines (appears not to listen vs. appears to listen), 4. Health review covers (high blood pressure only vs. high blood pressure and review of overall health)	attention paid by professional to your views about medicines	process
7	Gerard et al.	2014	England	Nurse-led vs. Doctor-led primary care: identify and quantify patient preferences for both professions of prescribers and factors that influence choice of who to consult	1. Access (next day at surgery (NIP) vs. same day at WiC (NIP)/2 days later at surgery (doc) vs. next day at surgery (doc)) 2. Length of consultation (10/20/30/40 min (NIP) vs. 5/10/15/20 min (doc)), 3. Professional’s attention paid to your views on your problem/medicines (appears not listen vs. appears to listen), 4. ‘help offered’ (only advice provided vs. diagnosis and advice provided)	professional’s attention paid to your views on your problem/medicines	process
8	Hjelmgren, Anell	2007	Sweden	Models/provider of primary care: examine which attributes are important when individuals choose between primary care models	1. Primary care work model (GP vs. primary care team), 2. Waiting time for non-emergency visits (2 days/4 days/7 days), 3. User charges (0 SEK/ 100 SEK/200SEK/300SEK), 4. Ability to choose provider (individual choice of provider (GP or team) vs. no choice), and 5. Degree of influence over the care received (large influence vs. limited influence).	degree of influence over the care received	process
9	Hole	2008	England (Greater Manchester area)	Appointment booking: examine the preferences on the choice of GP appointments	1. Number of days wait for an appointment (same day/next day/2 days/5 days), 2. Cost of appointment to patient (£0,/£8/£18/£28) 3. Flexibility of appointment times (one appointment offered vs. choice of appointment times offered) 4. Physician’s interpersonal manner (warm and friendly vs. formal and businesslike), 5. Physician’s knowledge of the patient (the doctor has access to your medical notes and knows you well vs. the doctor has access to your medical notes but does not know you) 6. Thoroughness of physical examination (the doctor gives you a thorough examination vs. the doctor’s examination is not very thorough)	Thoroughness of physical examination	process
10	Lagarde et al.	2015	England	Primary care (consultations) in general: explore the determinants of the choice of practice registration (especially the possibility of registering outside a patient’s neighborhood)	1. Practice is open on Saturday & Sunday (yes vs. no) 2. Practice is open at lunchtime (yes/never/sometimes), 3. Extended opening hours (yes vs. No), 4. How quickly you can normally be seen by a GP (same day/next day/a few days later/a week or more), 5. Whether the practice meets your specific health needs (yes vs. no), 6. How well the practice knows the health care services (previous experience with most of the health care providers in your neighborhood vs. no previous experience)	how quickly you can normally be seen by a GP	structure
11	Mengoni et al.	2013	Italy (Tuscany region)	Primary care (consultations) in general: assess patients’ preferences for different attributes of GP consultation	1. Waiting time for the visit (0 min/90 min/180 min), 2. Involvement in decision making (complete/partial/no), 3. Amount of information (a lot of information/some information/a little information)	amount of information	process
12	Pedersen et al.	2012	Denmark	Primary care (consultations) in general: investigate whether general practitioners know patients’ preferences regarding a number of organizational characteristics in general practice	1. Waiting time on the telephone (1 min/5 min/15 min/30 min), 2. Opening hours (no extended opening hours vs. open on Saturdays), 3. Waiting time to the appointment (same day/3 days/1 week/2 weeks), 4. Distance to the general practice (1 km/5 km /15 km/30 km), 5. Waiting time in the waiting room (5 min/10 min/20 min/30 min), 6. Consultation time (5 min/10 min/20 min/30 min), and 7. Whether the GP or assisting personnel performs routine tasks (GP vs. nurse)	waiting time to the appointment	structure
13	Philips et al.	2012	“a Western European city”	Out-of-hours service: reveal the decision criteria of patients in choosing out-of-hours services	1. Type of consultation (hospital emergency department/general practitioner cooperative/home visit by the general practitioner on duty/pediatrician), 2. Waiting time between first contact or call and consultation (< 30 min/30-90 min / > 90 min), 3. Information about health problem and therapy (doctor does not give enough information vs. doctor gives enough information), 4. Accessibility of the service (location and phone number are not known vs. location and phone number are known), 5. Availability of technical equipment (available vs. not available), 6. Method of payment (immediate payment vs. deferred payment)	information about health problem and therapy	process
14	Rubin et al.	2006	England (Sunderland)	Booking appointments: investigate patient preferences when making an routine appointment for a GP in different patient groups	1. Time to appointment (same day/within 48 h/4 days/10 days), 2. Choice of doctor (your choice of doctor/any available doctor), 3. Choice of time (your choice of time/at a specified time)	choice of doctor	structure
15	Seghieri et al.	2014	Italy	Models/provider of primary care: elicit patient preferences for different primary care models	1. Waiting time for visit (0 min/90 min/180 min), 2. Primary care provider (own GP/primary care team/another GP), 3. Diagnostic facilities (a lot of diagnostic facilities/some diagnostic facilities/a few diagnostic facilities)	primary care provider	structure
16	Tinelli et al.	2009	Scotland	Prescribing pharmacists: investigate patients’ preferences for an innovative combined prescribing-and-dispensing role for pharmacists in the management of drug therapies	1. Time spent travelling to and waiting in the surgery, consulting with the GP (0 min/30 min/50 min), 2. Time spent travelling to and waiting in the pharmacy, consulting with the pharmacist (0 min/20 min/40 min), 3. Chance of receiving the ‘best’ treatment (low/medium/high), 4. The amount of money you have to spend to get the drug (3£/7£/12£/20£)	chance of receiving the ‘best’ treatment	outcome
17	Tinelli et al.	2014	Germany, England and Slovenia	Primary care (consultations) in general: inform public health policy on patients’ priorities when choosing health care in Europe and compare patients’ preferences in different European countries	1. ‘Information’ received from the GP (rarely/sometimes/most of the times/always), 2. ‘Booking time’ (next day/1 week/2 weeks/3 weeks), 3. ‘Waiting time’ in the GP practice (10 min/20 min/30 min/40 min), 4. ‘Listened to’ (rarely/sometimes/most of the times/always), 5. Being able to receive the ‘best care’ available for their condition (rarely/sometimes/most of the times/always)	being able to receive the ‘best care’ available for their condition	outcome
18	Turner et al.	2007	England (Leicestershire and London)	Primary care (consultations) in general: estimate the relative importance to patients of continuity of care compared with other aspects of a primary care consultation	1. Who you consult (You consult a GP vs. a nurse), 2. Know & trust (who you do not know vs. who you know and trust), 3. Information about your medical history (has information about your full medical history vs. does not have information about your full medical history), 4. Waiting time (same day / 2 days/5 days/10 days)	information about your medical history	process

### Preferences for primary health care

The 18 studies are based on a wide range of attributes concerning primary health care. As diverse as the used attributes, so are the results of the preferences measures. Table 4 gives an overview of the study objectives, attributes, attribute levels and the most important attribute in each DCE (Table 4).

Based on the results of the regression analyses conducted in the identified studies the attribute “Care provider” was the most important in 4 of the DCEs [17, 18, 25, 28]. Caldow et al. [25] for example show that it is most important for respondents to see a GP rather than a practice nurse. This is even more important than “Continuity of health professional”, “Waiting time till appointment”, “Likelihood of having illness cured” and “Length of consultation”.^3^ Furthermore, 3 studies identified “Shared decision making” as being most important to the respective participants [19, 20, 30]. Hjelmgren and Anell [30] state that having influence on the decision of the care they receive is most important for respondents, followed by “Individual choice of GP/care provider”, “Waiting time till appointment” and “Price”. Other studies show that “Waiting time” is the most important attribute when choosing a primary care alternative [3, 22, 29, 32]. In their study Pedersen et al. [29] identify the typical waiting time until appointment (for routine tasks) to be most important for patients, for instance. This attribute is more important than “Distance to the practice”, “Waiting time on the telephone” and “Length of consultation”. The other discrete choice experiments determine “Information and explanation (on medicines/treatment/problem)” [4, 27], “Continuity of health professional/physician’s knowledge of the patient” [24, 28], “Receiving he ‘best’ treatment” [26, 33], “Thoroughness of physical examination” [21] and “Individual choice of GP/care provider” [23] as the most important attribute.

## Discussion

Overall, this systematic review of preference studies has identified a considerable number of attributes which effect the organization of primary health care. Moreover, the results of the studies show different preferences for primary health care. Overall 8 studies identify a structure attribute and 8 studies a process attribute as being most important for the respondents. In 2 studies the most significant attribute is an outcome attribute. However, it should be noted that those are absolute numbers, not relative ones. As there are 4 studies that use only structure attributes, all in all there is a lower chance for a process attribute to be the most important one. In addition, there are only 4 studies that include an outcome attribute, so that the chance for those ones to be most significant is even lower. Relative to the number of studies including the attribute dimension in question, a process attribute is most often the one of highest importance, i.e. the one with the highest ß-coefficient in the regression analysis.^4^

In general, the selection of attributes strongly depends on the aim of a study. If a study for example focuses on preferences for appointment booking systems or out-of-hours services, it is evident that the study uses rather structure attributes than process ones. However, what is remarkable is that only a few studies use outcome attributes. Those using outcome attributes have quite different study objectives, such as models of primary care or primary care consultations in general. In two of the four studies that use an outcome attribute it turns out to be the most important one. In both studies it is the same attribute, which is “Receiving best treatment”.

Comparing the attributes used in the studies and their results there does not seem to be a pattern according to the studys’ objective or origin. The diversity of the results may have different other reasons: The study results may particularly be biased for example by (1) the specific research question of the study or aim of the study or by (2) the selection procedure of the attributes and their levels.

Primarily, the chosen attributes and, thus, the preferences elicited by the DCE depend on the specific research question. Even if the study objective is the same, the precise issue might differ. Pedersen et al. [29] and Turner et al. [24] for example both aim to assess primary care consultations in general, but while the first assess preferences regarding different organizational characteristics, the latter estimate the relative importance of continuity of care compared to other aspects of primary care. Therefore, unsurprisingly, these two studies obtain different results regarding the preferences for primary health care. Pedersen et al. find the attribute “Waiting time” as being the most important one and Turner and colleagues ascertain the process attribute “Information and explanation” to be most significant. Their different research questions may cause a different selection of attributes and consequently different results although the study objectives are the same. In this context, a replication study using the same research question, the same attributes and levels as an existing DCE but comparing different regions and/or populations would be a useful addition to the literature.

Most of the studies followed the recommendations of for example the ISPOR or other authors concerning the selection procedure of the attributes and levels, which should be supported by evidence like literature reviews, qualitative research or other scientific methods. Although the process of selecting the relevant attributes is highly important for a preference study, there are no precise guidelines on how to translate the literature search or the previous qualitative work into the final attributes and their levels [34]. Amaya-Amaya and colleagues [8] state that an adequate set of attributes and choice contexts, in combination with variation in the attribute levels, is necessary. How this is composed remains unclear in the majority of publications and might be part of the interpretation of the researcher. Only one third of the identified studies claimed to use literature research to identify the relevant attributes and eight studies used qualitative studies. Four studies did not report on the identification of attributes and attribute levels at all. This indicates that not all studies meet the requirements of good research practice, named in different guidelines for conjoint analysis or DCE [11, 13, 35].

### Limitations

This review of the literature has some limitations. First, the results of a DCE can be influenced by reasons other than the research question or the attribute selection, which cannot all be discussed in this article. Those reasons might be the inclusion of an opt-out option [11, 36] or the description of the scenario. Gerard [17] for example included a “high worry scenario” and a “low worry scenario” in the Discrete Choice Experiment and the results show that seeing a doctor of choice (compared with nurse) is more important if patients are in a chronic, high worry condition than in an acute, low worry condition. Another factor that can influence the results of a DCE is the range of attribute levels. If the attribute “Waiting time until appointment” for example varies between no waiting time, 1 day and 2 days the DCE would – all other attributes and levels equal – probably lead to different results if the levels of this attribute were 1 day, 3 days and 5 days. Attribute levels should be chosen as realistically as possible and extreme values should be avoided [13, 35].

Furthermore, due to the exclusion of non-English language articles, some relevant studies might not have been included in this review. Furthermore, it is possible that the use of other search terms would have led to other search results.

Finally, because the results of a DCE depend on various factors such as research question, selection of attributes and levels and also on method of data analysis, it is not possible to directly compare their results.

## Conclusion

This review gives an overview over the attributes used in DCE studies that measure preferences for primary care. The results of discrete choice experiments are quite diverse and difficult to compare with each other, because they depend on many different factors, such as the research question, the process of selecting relevant attributes or the method of analysis. In order to achieve a high comprehensibility it is important to transparently report all steps undertaken in a DCE as well as the interim results – especially of the literature research and the qualitative pilot study. This could, for example, be done by publishing the results of the literature search or the qualitative work, or by adding them to an (electronic) appendix. As the final selection of the attributes out of the range of all possible attributes is inherently less transparent and rather interpretative and driven by different interests, the prior steps should be documented as clearly and reproducibly as possible.

Furthermore, this review can be helpful for researchers planning to conduct a DCE in the field of primary health care because it gives a broad overview of attributes and levels used in DCEs in the past 10 years. It also highlights which attributes and dimensions of care provision are important to patients.

Although the results of the DCE studies are not directly comparable, DCEs generally give relevant information on patient preferences within the context of the study setting and can support political decisions that take into account the patients’ perspective.

## Additional files

Additional file 1: Systematic Review Protocol. (DOC 31 kb)

Additional file 2: Search strategy. (DOC 34 kb)

## Abbreviations

DCE: Discrete choice experiment
GP: General practitioner
ISPOR: International Society for Pharmacoeconomics and Outcomes Research
OECD: Organization for Economic Co-operation and Development
RUT: Random utility theory
WHO: World Health Organization

## Footnotes

1 Please note that there are two publications that base on the same DCE and present the same results. Although we identified 19 publications, we consider only the results of 18 DCE studies.

2 The attribute “Care provider/Care setting” only refers to the choice of a medical doctor versus a nurse practitioner or practice nurse. It does not relate to the individual choice of a known versus unknown GP.

3 It must be noted that “Care provider” (being seen by a GP rather than by a nurse) is more important than a unit change in the continuous attributes “Waiting time”, “Likelihood of having illness cured” and “Length of consultation”; which means more important than 1 day reduction in waiting time, 1% change in chance of having illness cured and 1 min increase in the length of consultation.

4 For an overview of attributes identified in qualitative studies on preferences for primary care, see for example Cheraghi-Sohi et al. [2].
