# Supplementary material for: Patients’ preferences for primary health care – a systematic literature review of discrete choice experiments
Source: BMC Health Serv Res. 2017 Jul 11;17:476. doi: 10.1186/s12913-017-2433-7 (PMC5505038; doi:10.1186/s12913-017-2433-7)
Supplement: Supplementary file 1 — Systematic Review Protocol. (DOC 31 kb) [file 12913_2017_2433_MOESM1_ESM.doc]

**“Patients’ Preferences for Primary Health Care – A Systematic Literature Review of Discrete Choice Experiments” – Systematic Review Protocol**

Authors: Kim-Sarah Kleij, Ulla Tangermann, Volker Amelung, Christan Krauth

**Background**

Primary care is a key element of health care systems [1] that addresses the population’s main health problems and often serves as the patients’ first level of contact with the health care system. As most of the Western populations are continuously aging and, hence, the burden of chronic diseases is increasing, primary care even gains in importance. Because many countries currently face a shortage of general practitioners in rural areas, the maintenance of an adequate primary care provision is a central task of health care systems and therefore highly important for health policy makers [2, 3]. In order to guarantee an adequate, needs-based medical supply strategies, such as new and innovative models of care are needed. As a consequence, it is of relevance for the future organization of primary care provision to know patients’ preferences for different aspects of primary care. The discrete choice experiment is a common and frequently used technique to elicit preferences for health care services, therapies, or technologies. Discrete choice experiments are an attribute-based method of measuring preferences that describe health services in terms of varying attributes and attribute levels. Thus, the identification of appropriate attributes and levels is an essential task of the study [4]. To examine the preferences for primary care, one should to consider which attributes (and levels) were included and how they were selected because they strongly affect a DCE’s results.

**Review questions**

This systematic review seeks to have a closer look at studies using a discrete choice experiment to elicit patients’ or the population’s preferences in a primary care setting. The specific review questions are:

(1) Which are the attributes and attribute levels used in DCEs for measuring preferences for primary care and how were they selected?

(2) Which attributes of primary care are most important for patients and the population within the scope of the identified DCE studies?

This review questions should be answered through the available literature.

**Inclusion criteria**

This review will consider discrete choice experiments published in English during the past ten years (2006-2015), which focus on general aspects of primary health care. Study participants will be either a sample of patients or the population. There’ll be no constraints regarding age, sex or origin of the study participants. All types of survey methods will be included, covering face-to-face, telephone, postal, and online surveys.

**Exclusion criteria**

Studies will be excluded if they were not conducted in an OECD country. This criteria should guarantee a better comparability of the health standards within the health care system. Furthermore, studies which solely focus on specific health conditions will be excluded because the objective was to cover a broad range of relevant attributes to all patients, not only patients with specific diseases. Also studies that exclusively address end-of-life or palliative care, or shared-decision making will be excluded.

**Search strategy**

The search strategy is supposed to identify publications based on discrete choice experiments, measuring preferences for primary health care. The initial search terms will be:

- "patient* preference*" OR "public preference*" OR "discrete choice" OR "DCE"
- "primary care" OR "general practitioner*" OR "GP*" OR "family doctor*" OR "family physician*" OR "family medicine*"

The search will be conducted in the following databases:

- PubMed
- Scopus
- PsycINFO

Additionally, a hand search will be performed in the reference lists of the included publications.

Full copies of publications identified by means of the search strategy and considered to meet the defined inclusion criteria, based on (1) title, (2) abstract, and (3) full text will be obtained for data analysis. Two reviewers will independently screen the titles, abstracts and full texts identified through the search.

**Data extraction and outcomes**

Information on authors, title, year of publication, country, source, and content/abstract will be extracted from the databases. Duplicates will be deleted. Afterwards the two reviewers will independently screen the extracted data for relevance and discuss ambiguous cases. The final identified studies will be screened in terms of the following outcomes:

- Study objective
- Study population
- (Number of) attributes and attribute levels used in the DCE
- Methods used to identify relevant attributes and levels
- Most important attribute (identified by results of data analysis, e.g. regression analysis)

**Data synthesis**

Because the results of a discrete choice experiment strongly depend on the selected attributes and levels, as well as on the methods of data analysis a direct comparison of the results (e. g. in terms of regression coefficients) probably won’t be possible. For that reason, data will be compared descriptively and summed up. This will at least be done for attributes included in the studies to give an overview of relevant aspects for the provision of primary health care.

**References**

1. WHO. Declaration of Alma-Ata. International Conference on Primary Health Care. Alma

Ata, USSR: World Health Organization 1978, 6-12.

2. Simoens S, Hurst J. The Supply of Physician Services in OECD Countries. OECD Health

Working Paper No. 21. 2006, Available at: http://www.oecd.org/health/health

systems/35987490.pdf.

3. Organization for Economic Co-operation and Development (OECD). Health at Glance:

Europe 2012, OECD Publishing 2012, Paris. http://dx.doi.org/10.1787/9789264183896-29-en.

4. Cheraghi-Sohi S, Bower P, Mead N, et al. What are the key attributes of primary care for

patients? Building a conceptual ‘map’ of patient preferences. Health Expectations 2006
