# Supplementary material for: Patients’ preferences for primary health care – a systematic literature review of discrete choice experiments
Source: BMC Health Serv Res. 2017 Jul 11;17:476. doi: 10.1186/s12913-017-2433-7 (PMC5505038; doi:10.1186/s12913-017-2433-7)
Supplement: Supplementary file 2 — Search strategy. (DOC 34 kb) [file 12913_2017_2433_MOESM2_ESM.doc]

Additional file 2: Search strategy

| #1 | „patient preference*“ |
| --- | --- |
| #2 | "patient priorities" |
| #3 | "public preference*" |
| #4 | #1 OR #2 OR #3 |
| #5 | "discrete choice" |
| #6 | "DCE" |
| #7 | "stated preference*" |
| #8 | "conjoint analysis" |
| #9 | #5 OR #6 OR #7 OR #8 |
| #10 | #4 OR #9 |
| #11 | "primary care" |
| #12 | "general practitioner*" |
| #13 | "GP*" |
| #14 | "family doctor*" |
| #15 | "family physician*" |
| #16 | "family medicine*" |
| #17 | #11 OR #12 OR #13 OR #14 OR #15 OR #16 |
| #18 | #10 AND #17 |
